# Molecular Detection of *Alpha*‐ and *Betacoronaviruses* in Bats from Afar Region, Ethiopia

**DOI:** 10.1002/vms3.70808

**Published:** 2026-04-06

**Authors:** Bula Mengesha, Gebremedhin Romha, Gebremedhin Gebrezgabiher

**Affiliations:** ^1^ Ministry of Agriculture Addis Ababa Ethiopia; ^2^ Department of Veterinary Public Health and Food Safety College of Veterinary Sciences Mekelle University Mekelle Ethiopia; ^3^ Department of Veterinary Medicine College of Veterinary Medicine and Animal Sciences Samara University Samara Ethiopia

**Keywords:** Afar, bats, coronavirus, Dire Dawa, Ethiopia

## Abstract

**Background:**

Bats are critical reservoirs of coronaviruses, including zoonotic strains, yet gaps remain in understanding coronavirus diversity within Ethiopia's understudied bat populations. This study investigated coronavirus prevalence and genetic diversity in bats from Afar and Dire Dawa, Eastern Ethiopia.

**Methods:**

A cross‐sectional study was conducted from November 2015 to June 2016 in purposively selected sites of Afar and Dire Dawa, Ethiopia, where bats were captured using 12 × 3‐m mist nets. Ribonucleic acid (RNA) was screened by real‐time polymerase chain reaction (RT‐PCR) targeting the RNA‐dependent RNA polymerase (RdRp) gene, with confirmatory PCR for the open reading frame 1a (ORF1a) region.

**Results:**

A total of 141 bats were captured, including 129 *Chaerephon pumilus*, 11 *Epomophorus labiatus* and 1 *Mops condylurus*, from which 278 oropharyngeal swabs, rectal swabs, and urine specimens were collected. Coronaviruses were detected in eight bats (5.7%): five *C. pumilus*, two *E. labiatus* and one *M. condylurus*. Of these, three were detected from rectal swabs, three from oral swabs and two from urine samples. A higher proportion of adult bats (6.7%, 6/89) carried the virus compared to juveniles, with similar proportions of males (5.5%, 3/55) and females (5.8%, 5/86) testing positive. Sequencing revealed six *Alphacoronaviruses* (75%) and two *Betacoronaviruses* (25%; lineage D).

**Conclusion:**

The study contributes important baseline data on coronavirus diversity in the area. Continued genomic surveillance, including full‐genome sequencing and broader ecological sampling, is essential to advance understanding of coronavirus evolution and maintenance within bat populations.

## Graphical abstract

Bats are critical reservoirs of coronaviruses, yet gaps remain in understanding coronavirus diversity within bat populations in Eastern Ethiopia. A total of 141 bats were captured: 129 *Chaerephon pumilus*, 11 *Epomophorus labiatus* and 1 *Mops condylurus. Coronaviruses* detected in eight bats. The study contributes baseline data to the understanding of coronavirus diversity.

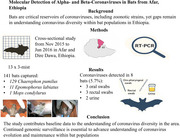

**Abbreviations**
Bp: base pair
MERS: Middle East respiratory syndrome
ORF1a: open reading frame 1a
PCR: polymerase chain reaction
PEDV: porcine epidemic diarrhoea virus
RT‐qPCR: reverse transcription‐quantitative polymerase chain reaction
SARS: severe acute respiratory syndrome
UpE: upstream of the Envelope gene
VTM: viral transport media

## 1 Background

Coronaviruses are important pathogens of both animals and humans (Wong et al. 2019). They are classified into four genera—*Alphacoronavirus*, *Betacoronavirus*, *Gammacoronavirus* and *Deltacoronavirus*—based on their genetic and antigenic characteristics (Chu et al. 2011; Tao et al. 2017). Several coronaviruses circulate widely in animal reservoirs, particularly in wildlife, and some have demonstrated the capacity to cross species barriers and establish infection in humans. Such spillover events have produced human coronaviruses responsible for mild respiratory illnesses as well as severe and sometimes fatal diseases, such as severe acute respiratory syndrome (SARS), Middle East respiratory syndrome (MERS) and, most recently, COVID‐19. These include the following: (i) SARS‐coronavirus: a *Betacoronavirus* that emerged in 2002 (Shi and Wang 2017); (ii) MERS‐CoV, identified in 2012 and associated with sporadic but severe outbreaks (Memish et al. 2013); and (iii) SARS‐CoV‐2, the causative agent of the global COVID‐19 pandemic (Wu et al. 2020).

Bats are now recognized globally as major reservoirs of diverse coronaviruses. Previous studies in China revealed SARS‐related coronaviruses in horseshoe bats and several novel *Alphacoronaviruses* in other species (Lau et al. 2005; Poon et al. 2009; Aris et al. 2005). Subsequent large‐scale surveillance in Asia and the Americas further demonstrated remarkable coronavirus diversity: for example, multiple genetically *Alpha*‐ and *Betacoronaviruses* in China (Lau et al. 2005; Poon et al. 2009; Tang et al. 2006; Li et al. 2005), novel *Alphacoronaviruses* in Colombia (Martínez et al. 2025), 13 distinct coronaviruses (9 *Alpha*‐ and 4 *Betacoronaviruses*) in bats from Mexico (Anthony et al. 2013) and 47 coronaviruses (37 *Alphacoronaviruses* and 10 *Betacoronaviruses*) across 13 different bat species in Eastern Thailand (Wacharapluesadee et al. 2015). In Africa, studies from Kenya have documented both novel coronaviruses and lineages closely related to the human coronaviruses NL63 and 229E (Tao et al. 2017), underscoring the continent's reservoir potential.

Despite Africa's rich chiropteran diversity, Ethiopia remains one of the least studied countries regarding bat‐associated coronaviruses. Although over 80 bat species occur in the country (Ahmed et al. 2024), the diversity of coronaviruses circulating within these populations is still largely unknown. To date, only one published study has reported coronavirus detection in Ethiopian bats (Lane et al. 2022). However, the study was geographically limited and did not provide broader insights into the diversity, distribution and phylogenetic relationships of coronaviruses circulating among different Ethiopian bat species. This study addresses this gap by expanding coronavirus surveillance to additional ecological sites across Afar and Dire Dawa in Eastern Ethiopia. By identifying and characterizing coronaviruses circulating among bats in these regions, the study provides essential baseline data needed to understand the country's viral diversity, potential zoonotic risks and priority areas for future surveillance.

## 2 Materials and Methods

### 2.1 Study Setting and Design

**FIGURE 1:**
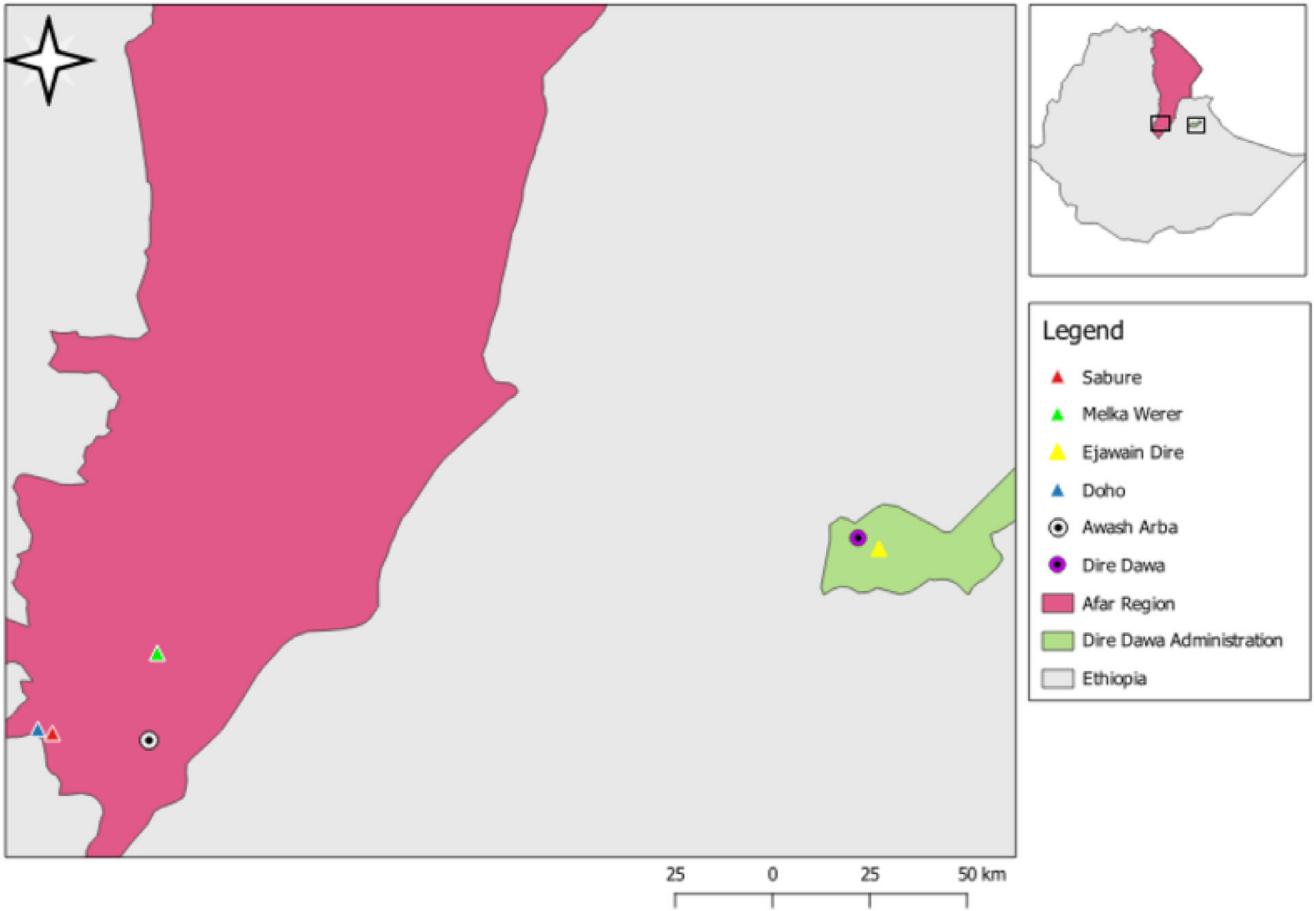
Map of the study areas and bat capture sites.

A cross‐sectional study was conducted from November 2015 to June 2016 in selected *kebeles* (the smallest administrative units) of the Afar Region and the Dire Dawa Administration in Eastern Ethiopia to investigate coronavirus prevalence and diversity among local bat populations. The study *kebeles*—Doho, Melka Werer and Sabure in the Afar Region, and Ejawain in Dire Dawa—were purposively selected on the basis of preliminary field data indicating bat presence (Figure 1). The Afar Region is located in Northeastern Ethiopia, covering approximately 270,000 km^2^, with elevation ranging from 120 to 1500 m above sea level. The climate is predominantly arid to semi‐arid, with bimodal rainfall (150–500 mm/year) and mean monthly temperatures between 20°C and 48°C. Pastoralism is the primary livelihood, practiced by 90% of the population (CSA 2007). Dire Dawa has elevations between 960 and 2450 m. Mean annual rainfall ranges from 550 to 850 mm, and mean monthly temperatures range from 14.5°C to 21.6°C (DDAEPA 2020).

### 2.2 Bat Capture Protocol and Identification

**FIGURE 2:**
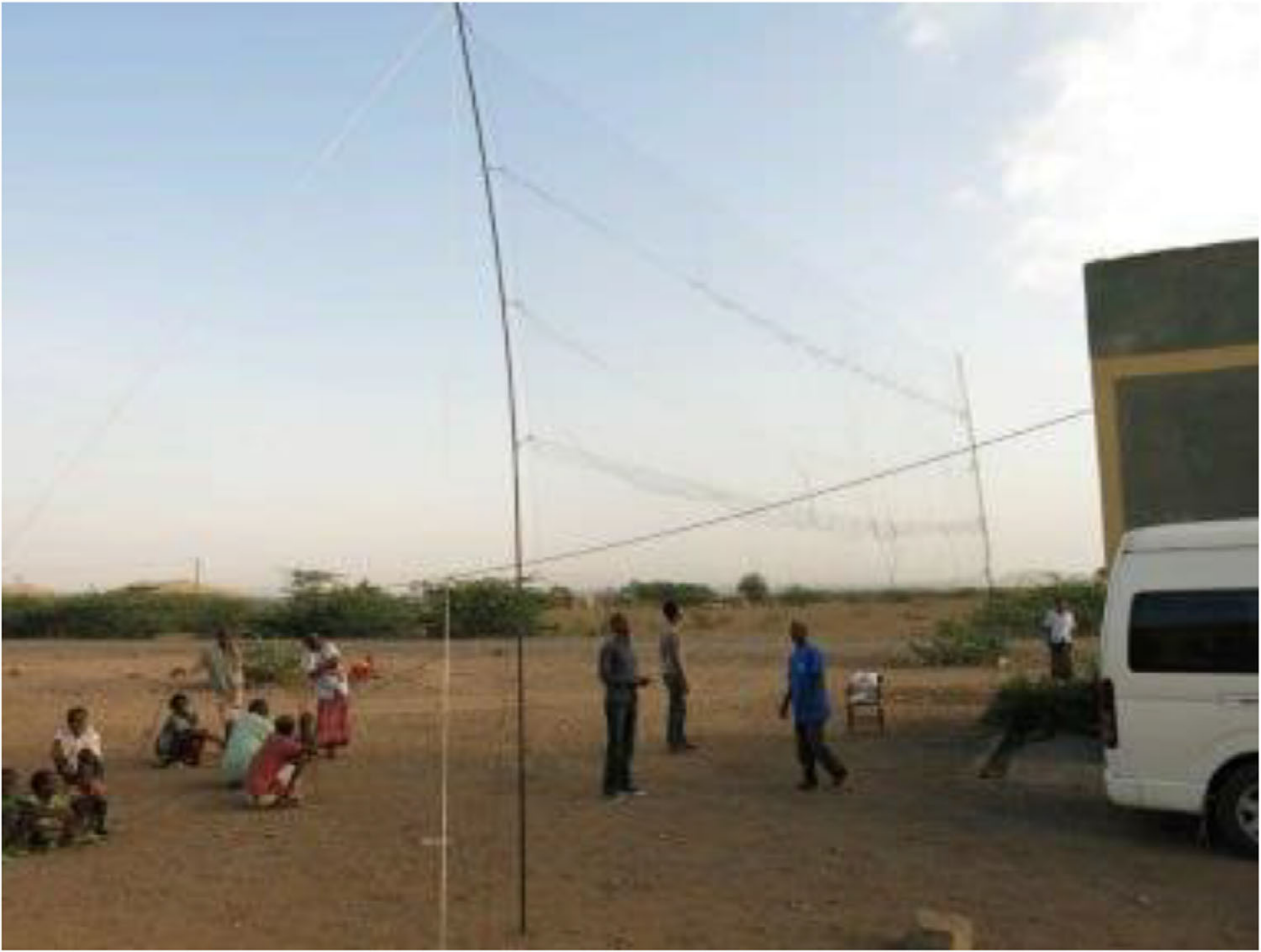
Protocol of bat capture in the study areas using the mist nets.

All bats were captured using 12 × 3‐m mist nets (Figure 2). The study was conducted during the cold, dry season, a period when most fruit trees were barren, making it unsuitable for capturing *Pteropus* (fruit bats). Consequently, the focus was primarily on *Microchiroptera* (small, echolocating bats) that roost in caves, trees and buildings (such as house roofs). Mist nets were strategically set up near buildings with bat populations in the study Kebeles. Then, each captured bat was identified morphologically using the identification matrix for Southern African Chiropteran families (Monadjem et al. 2020).

### 2.3 Specimen Collection

We collected two oropharyngeal and two rectal swabs from each captured bat, storing them in viral transport media (VTM). Urine samples were collected using swabs when bats urinated during handling and were also placed in VTM. Oropharyngeal swabs were obtained from all captured bats. Rectal swabs were easily collected from larger bats, *Epomophorus labiatus* and *Mops condylurus*. For bats weighing less than 8 g, rectal swabs were only collected when the bats defecated during handling (faeces on swab). All samples were stored in cooler boxes with ice packs and refrigerated until they reached Bishoftu, where they were transferred to and stored in a −20°C deep freezer. The molecular detection was done at the University of Hong Kong, China.

### 2.4 Ribonucleic Acid (RNA) Extraction and Polymerase Chain Reaction (PCR)

Total nucleic acid was extracted from swab samples using the NucliSENS easyMAG system (bioMérieux) following the manufacturer's instructions, with an extraction negative control included in each batch. Initial coronavirus screening used the broadly reactive RT‑PCR assay targeting a conserved region of the RNA‐dependent RNA polymerase (RdRp) gene as previously described (Poon et al. 2009). This assay amplifies a ∼440 base pair (bp) fragment of the RdRp region that is highly conserved across known *Alpha*‐ and *Betacoronaviruses* and is widely used for wildlife coronavirus surveillance. The real‐time polymerase chain reaction (RT‐PCR) was conducted using a one‐step RT‐PCR kit with the following thermocycling conditions recommended by Poon et al. (2009): reverse transcription at 50°C for 30 min; initial denaturation at 95°C for 3 min; 40 cycles of 95°C for 15 s, 50°C for 30 s and 72°C for 45 s; and a final extension at 72°C for 5 min. Each run included a no‐template control, an extraction negative control and a positive control consisting of archived coronavirus‐positive RNA from the collaborative reference laboratory. All RdRp‐positive specimens were further confirmed using a probe‐based second RT‐qPCR assay targeting the open reading frame 1a (ORF1a) region of the genome, following the TaqMan protocol established by Poon et al. (2009). Thermocycling conditions for the confirmatory assay were reverse transcription at 50°C for 10 min, polymerase activation at 95°C for 2 min, followed by 45 cycles of 95°C for 15 s and 60°C for 1 min. For genomic characterization, the Spike glycoprotein 2 (S2) regions of the positive samples were amplified and sequenced following the protocol and primer sets described by Chu et al. (2006). The S2 subunit contains phylogenetically informative motifs that allow discrimination among coronavirus lineages and has been used in prior bat coronavirus studies for lineage placement. Amplicons were generated using standard PCR conditions (95°C for 3 min; 35 cycles of 95°C for 15 s, 52–58°C for 30 s and 72°C for 60 s; final extension at 72°C for 5 min). In addition, the full spike gene and a 400‐bp fragment of the ORF1b of one virus were sequenced to enable deeper evolutionary comparison (Chu et al. 2006).

### 2.5 Data Analysis

Data entry, cleaning, summarization and visualization were performed using Microsoft Excel and SPSS version 20. Descriptive statistics were used to summarize prevalence and animal characteristics, sample type and bat capture sites. The nucleotide data from diagnostic sequencing of the RdRp fragment were compared with existing coronavirus sequences in GenBank to evaluate the diversity of the detected coronaviruses. A phylogenetic tree was constructed using a 400‐nucleotide region of the RdRp gene in ORF1b, using the PhyML software based on the maximum likelihood method.

### 2.6 Ethical Considerations

The study was approved by the research and publications committee of Dilla University (Reference number: RERC011/15). The permission to capture the bats and collect the specimens was obtained from the local authorities. The research team kept the welfare of the bats during the study and strictly adhered to ethical standards and international guidelines for animal handling and sampling. All necessary precautions were taken to ensure the bats’ safety and well‐being during capture, handling and sampling procedures. The captured bats were released following the sampling procedures.

## 3 Results

### 3.1 Captured Bat Species and Collected Samples

**FIGURE 3:**
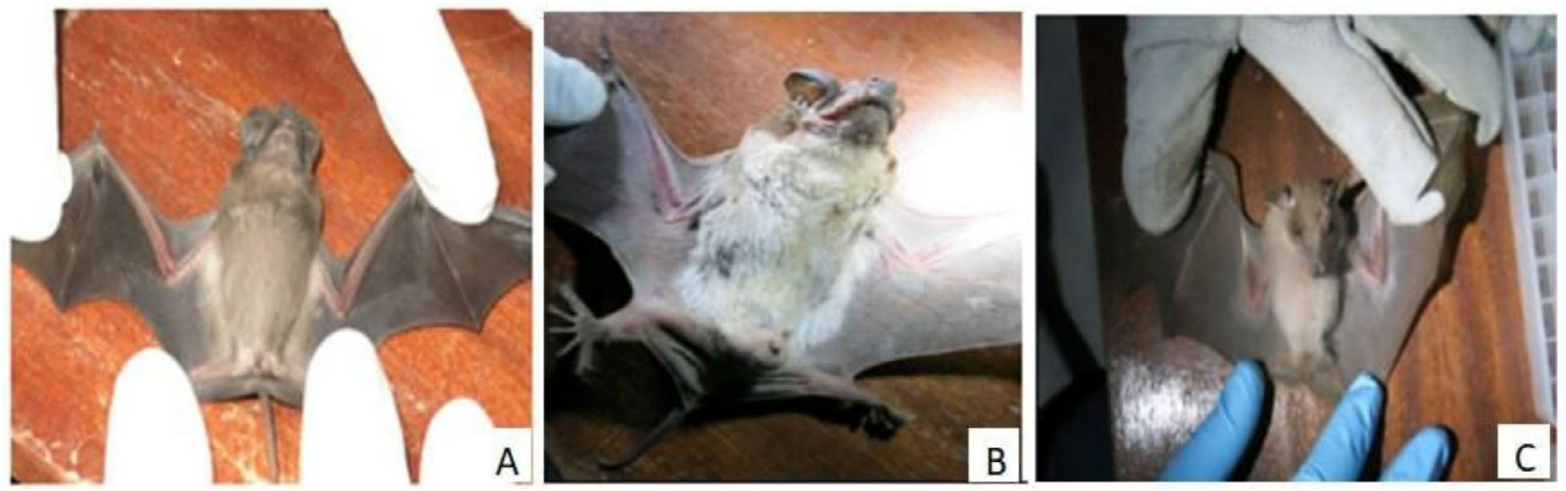
Bat species captured during the study: (A) *Chaerephon pumilus*; (B) *Mops condylurus* and (C) *Epomophorus labiatus*.

A total of 141 bats representing three species were captured across four sites: 129 *Chaerephon pumilus*, 11 *E. labiatus* and 1 *M. condylurus* (Figure 3). Most captures occurred in Melka Werer, Doho and Sabure, with fewer bats obtained in Ejawain. Females accounted for 61% of sampled bats, and 64.5% were adults. In total, 278 specimens were collected, consisting of 141 oropharyngeal swabs, 99 rectal swabs and 38 urine samples. Table 1 summarizes species distribution, sex and age composition, sampling site and specimen collected.

**TABLE 1:** Captured bat species and their sex and age composition, respective collection sites and specimen type collected.

		Bat species			
Variable	Category	*Chaerephon pumilus n* (%)	*Epomophorus labiatus n* (%)	*Mops condylurus n* (%)	Total *n* (%)
Sex	Male	1 (1.8)	53 (96.4)	1 (1.8)	55 (39)
	Female	10 (11.6)	76 (88.4)	0 (0)	86 (61)
Age	Juvenile	0 (0)	50 (100)	0 (0)	50 (35.5)
	Adult	11 (12.1)	79 (86.8)	1 (1.1)	91 (64.5)
Site	Melka Werer	34 (75.6)	11 (24.4)	0 (0)	45 (31.9)
	Doho	49 (100)	0 (0)	0 (0)	49 (34.8)
	Sabure	39 (97.5)	0 (0)	1 (2.5)	40 (28.4)
	Ejawain	7 (100)	0 (0)	0 (0)	7 (5)
Specimen	Oropharyngeal swab	129 (91.5)	11 (50.4)	1 (0.7)	141 (50.7)
	Rectal swab	87 (87.9)	11 (11.1)	1 (1.01)	99 (35.6)
	Urine	37 (97.4)	0 (0)	1 (2.6)	38 (13.7)

### 3.2 Prevalence of Bat Coronaviruses

Out of 141 bats captured and sampled, 8 (5.7%; 95% CI: 1.8–9.5) tested positive for coronaviruses. Specifically, 4% (5/129) of *C. pumilus* and 18.2% (2/11) of *E. labiatus* were positive, along with the single *M. condylurus* sampled. Virus prevalence was similar between male (5.5%, 3/55) and female (5.8%, 5/86) bats. However, adults showed a higher rate of infection compared to juveniles. Most positive detections occurred in Sabure (62.5%, 5/8), followed by Melka Werer (25%, 2/8) and Doho (12.5%, 1/8). All the 278 samples were screened individually, and coronavirus detection differed by specimen type, with positivity rates of 2.1% (3/141) in oropharyngeal swabs, 3.03% (3/99) in rectal swabs and the highest rate of 5.3% (2/38) observed in urine samples (see Table 2).

**TABLE 2:** Bat coronavirus prevalence by sex, age, species and specimen type sampled.

Variable (s)	Category	Bats captured (%)	Positive *n* (%)
Sex	Male	55 (39)	3 (5.5)
	Female	86 (61)	5 (5.8)
Age	Juvenile	50 (35.5)	2 (4)
	Adult	91 (64.5)	6 (6.6)
Bat species	*Epomophorus labiatus*	11 (7.8)	2 (18.2)
	*Chaerephon pumilus*	129 (91.5)	5 (3.9)
	*Mops condylurus*	1 (0.7)	1 (100)
Capture site	Melka Werer	45 (31.9)	2 (4.4)
	Sabure	40 (28.3)	5 (12.5)
	Doho	49 (34.8)	1 (2)
	Ejawain	7 (5)	0 (0)
Specimen type	Oropharyngeal swab	141 (51.1)	3 (2.1)
	Rectal swab	99 (35.6)	3 (3)
	Urine	38 (13.7)	2 (5.3)

In *C. pumilus*, *Alphacoronaviruses* were detected in 1.6% (2/129) of oropharyngeal swabs, 1.1% (1/87) of rectal swabs and 5.4% (2/37) of urine samples. In *E. labiatus*, no viruses were found in the 11 oropharyngeal swabs, whereas 18.2% (2/11) of rectal swabs were positive for *Betacoronaviruses*; no urine samples were collected for this species. For *M. condylurus*, the only oropharyngeal swab collected tested positive (100%) for an *Alphacoronavirus*, whereas the single rectal and urine samples were negative. No cases of co‐infections were detected. Table 3 summarizes the number of specimen types collected from each bat species and the corresponding positive samples.

**TABLE 3:** Type of samples collected from the respective bat species and their coronavirus isolates.

	Oropharyngeal swab		Rectal swab		Urine	
Bat species	Total	Positive (%)	Total	Positive (%)	Total	Positive (%)
*Chaerephon pumilus*	129	2 (1.6)^α^	87	1 (1.1)^α^	37	2 (5.4)^α^
*Epomophorus labiatus*	11	0	11	2 (18.2)^β^	—	—
*Mops condylurus*	1	1 (100)^α^	1	0 (0)	1	0 (0)

*Note*: α: *Alphacoronavirus*; β: *Betacoronavirus*.

### 3.3 Phylogenetic Analysis of Coronaviruses

**FIGURE 4:**
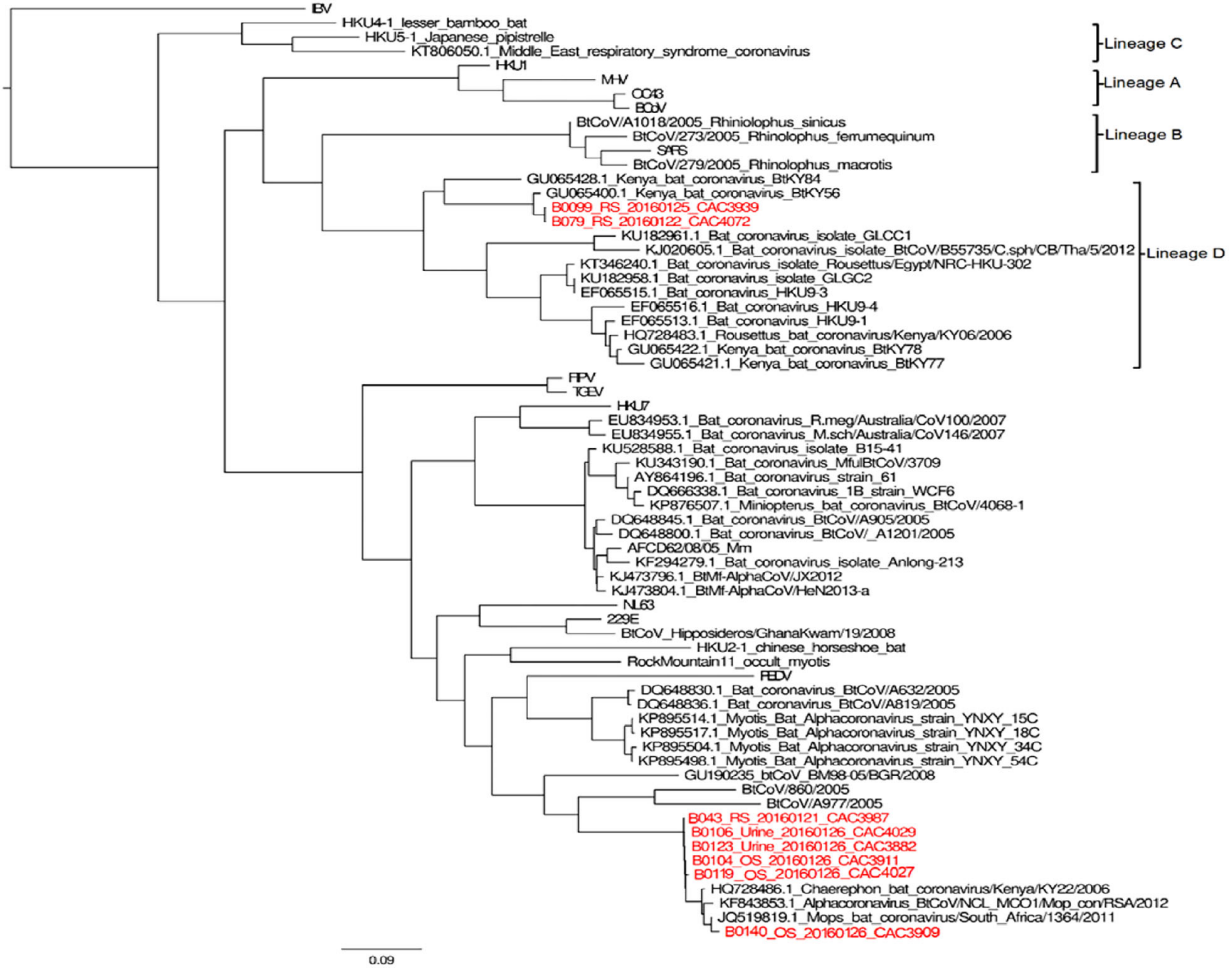
Maximum‐likelihood phylogenetic tree of partial RdRp gene sequences (440 bp) illustrating the evolutionary relationships between coronavirus strains detected in this study (shown in red and named following sample name, sample type, sample collection date and laboratory number) and representative GenBank reference sequences. Phylogenetic reconstruction was performed in PhyML using the GTR + G substitution model, with branch‐support values estimated from 1000 bootstrap replicates. Major coronavirus lineages, including *Alpha‐* and *Betacoronavirus* lineages A–D, are indicated. The Ethiopian bat coronavirus sequences cluster within both *Alpha*‐ and *Betacoronavirus* clades, grouping alongside previously reported bat coronaviruses. The scale bar denotes nucleotide substitutions per site, and the topology highlights the genetic diversity of coronaviruses circulating in the sampled bat populations and their relatedness to globally characterized strains.

A total of eight coronavirus strains were identified using a 400 bp fragment of the RdRp gene (ORF1b). Two strains (25%; samples B0099 and B079) clustered within the *Betacoronavirus* lineage D, whereas the remaining six strains (75%) grouped within the *Alphacoronavirus*es. The two *Betacoronaviruses* were detected in rectal swabs from *E*. *labiatus*. Among *Alphacoronaviruses*, five strains were detected in *C. pumilus* (two from oral swabs, two urine samples and one rectal swab) and one strain in *M. condylurus* (oral swab). See Figure 4.

## 4 Discussion

This study provides the first molecular evidence and phylogenetic characterization of both *Alpha*‐ and *Betacoronaviruses* circulating in bat populations in the Afar region, Ethiopia. The overall coronavirus prevalence of 5.7% aligns with detection rates reported in similar surveillance studies from Uganda (Anthony et al. 2017) and China (Tang et al. 2006), though it remains lower than estimates from Germany (9.8%) (Gloza‐Rausch et al. 2008) and parts of China, where prevalence exceeded 15% (Chu et al. 2006; Lau et al. 2020). Such variations across studies likely reflect differences in sampling intensity, seasonality, bat community composition and assay sensitivity, all of which influence coronavirus detection in wildlife populations.

In this study, *Alpha*‐ and *Betacoronaviruses* were detected in *C. pumilus*, *E. labiatus* and *M. condylurus*, consistent with global patterns showing that diverse bat taxa host long‐established coronavirus lineages (Wong et al. 2019; Anthony et al. 2013; Wacharapluesadee et al. 2015; Hashemi‐Shahraki et al. 2013). The identification of *Alphacoronaviruses* in the *C. pumilus* and *M. condylurus* aligns with their known ecological roles and the global predominance of *Alphacoronaviruses* among insectivorous bats (Hu et al. 2017). These detections reflect deep host–virus associations shaped by long‐term co‐evolution, a process that likely promotes stable viral maintenance within bat populations rather than frequent cross‐species transmission. Similar observations from Ethiopia (Lane et al. 2022) further indicate that local bat communities support diverse coronavirus assemblages shaped by ecological networks, roosting dynamics and evolutionary history.

The detection of viral RNA in oropharyngeal swabs, rectal swabs and urine samples from *C. pumilus* suggests that multiple excretion routes may be involved in viral shedding. Although faecal shedding is often dominant in African bat coronaviruses (Corman et al. 2015), the multi‐route detection observed here may reflect variation in tissue tropism across coronavirus lineages or differences in infection stage among individuals. However, given the limited number of positives, these findings should be interpreted cautiously. More targeted sampling, including longitudinal monitoring, is needed to determine whether shedding routes vary seasonally or among sympatric bat species.

In this study, the detection of a *Betacoronavirus* lineage D in *E. labiatus* is noteworthy from an evolutionary perspective, as African bats have previously been shown to harbour diverse *Merbecovirus*‐related sequences (Memish et al. 2013; Hashemi‐Shahraki et al. 2013). Although this sequence shares a distant common ancestry with MERS‐CoV, the relatively low nucleotide similarity (∼51%–52%) observed in this study indicates substantial evolutionary divergence. Importantly, partial RdRp fragments do not permit inference of zoonotic potential. Thus, although the presence of lineage D *Betacoronaviruses* highlights the ecological richness of Ethiopian bat viruses, it does not imply direct epidemiological relevance to known human or livestock coronaviruses. Instead, these findings underscore the value of continued surveillance and full‐genome sequencing to better understand the evolutionary landscape of African bat coronaviruses.

Comparisons with previous studies showing near‐complete genomic similarity between some African bat coronaviruses and MERS‐related viruses (Memish et al. 2013; Corman et al. 2014) highlight the heterogeneity of evolutionary relationships across regions and bat hosts. Coronaviruses are known to undergo frequent recombination (Corman et al. 2015), enabling the long‐term diversification of lineages across bat species and geographic ranges. The phylogenetic affiliations observed here—with Ethiopian sequences clustering near viruses from Kenya and South Africa—likely reflect both deep co‐evolutionary patterns and ecological links through shared ancestry or historical bat dispersal across Eastern and Southern Africa.

In this study, coronavirus detections occurred in both male and female bats in similar proportions, consistent with other study reporting limited sex‐associated differences in prevalence (Gloza‐Rausch et al. 2008). Although adults showed a higher apparent prevalence than juveniles, the small sample size limits robust conclusions. Age‐related differences in immunity, diet or roosting behaviour could play a role and warrant further targeted investigation. The spatial clustering of positives in Sabure and Melka Werer may reflect local ecological or behavioural factors—such as high colony density, specific roost structures or resource availability—that facilitate viral persistence and intraspecies transmission (Plowright et al. 2014).

In this study, phylogenetic clustering of Ethiopian sequences with those in Kenya and South Africa (Hashemi‐Shahraki et al. 2013; Tong et al. 2009) further supports the concept of regionally interconnected coronavirus lineages maintained through long‐term host–virus co‐evolution, rather than recent long‐distance viral dispersal.

This study has several limitations that should be considered when interpreting the findings. First, sampling was limited to specific geographic areas and involved a relatively small sample size due to financial constraints, which may limit the generalizability and robustness of the findings, particularly when drawing broader ecological and evolutionary inferences. Second, the use of partial rather than whole‐genome sequences limited the study's ability to provide better insights into the evolutionary relationships and precluded stronger inferences regarding potential zoonotic risk. Third, the study did not assess ecological or behavioural traits of the sampled bat species—such as migratory patterns and interspecies interaction—which may influence viral circulation dynamics and would be valuable to integrate in future research.

## 5 Conclusions

This study contributes important baseline data on coronavirus diversity in the study area. Although the partial sequences limit inference on viral phenotypes or zoonotic potential, the findings highlight ecologically rich viral communities. Continued genomic surveillance, including full‐genome sequencing and broader ecological sampling, is essential to advance understanding of coronavirus evolution and maintenance within bat populations.

## Author Contributions

Bula Mengesha, Gebremedhin Romha and Gebremedhin Gebrezgabiher conceived the design of the study. Bula Mengesha collected the data. Bula Mengesha, Gebremedhin Romha and Gebremedhin Gebrezgabiher analysed the data, drafted wrote and approved the manuscript.

## Funding

This research was financially supported by Dilla University and the UNDP project. The MERS‐CoV Project provided technical support for bat capture and sample collections techniques, whereas Hong Kong University assisted with sample processing.

## Ethics Statement

The study was approved by the research and publications committee of Dilla University. The permission to capture the bats and collect the specimens was obtained from the local authorities. The research team maintained the welfare of the bats during the study and strictly adhered to ethical standards and international guidelines for animal handling and sampling. All necessary precautions were taken to ensure the bats’ safety and well‐being during capture, handling and sampling procedures. The captured bats were released after completion of the sampling procedures.

## Conflicts of Interest

The authors declare no conflicts of interest.

## Data Availability

The datasets used for this study are available from the corresponding author upon reasonable request.
